# Septic shock, noradrenaline requirements and alpha-2 agonists: Fishing in the right pond?

**DOI:** 10.1186/s13054-020-03377-5

**Published:** 2021-02-09

**Authors:** Auguste Dargent, Luc Quintin, Jean-Pierre Quenot

**Affiliations:** 1 Critical Care, CHU Dijon, Dijon, France; 2 Critical Care, Hôpital d’Instruction des Armées Desgenettes, Lyon, France

Bellomo et al. [1] confirm the clinical [2–4] data showing a reduction in noradrenaline (NA) requirements in the setting of septic shock following administration of an alpha-2 agonist, dexmedetomidine.

The intensivist is unaware that the retrospective analysis has thoroughly changed the design of the study from “sedation vs. outcome” to “sympathetic de-activation vs. circulation” (i.e., upregulation of alpha-1 receptors vs. NA requirements). Furthermore, the design is not optimal. SPICE III [5] compared early dexmedetomidine (“dex”) versus usual sedation (− 2 < RASS < + 1) [5]. Bellomo achieved RASS ~ − 4, in both groups (results [1]): The dex group received also propofol (95% of the patients), midazolam (43%) and *higher* doses of opioids (Table S1 [1]). Thus, any effect of dex is *drowned* as a consequence of adding usual sedation to dex. Nevertheless, in the dex group, (a) the overall NA requirement (“NA equivalent”) is lowered by 25%, nonsignificantly, but of daily clinical relevance for the intensivist; (b) the NA requirement necessary to achieve a target pressure is lowered, as a function of dose (i.e., compatible with a dose-dependent sympathetic de-activation). NA requirements should be readdressed in the dexmedetomidine-*only* patients versus the usual sedation-*only* patients, throughout the whole SPICE III [5] database.

## Data Availability

Not applicable.

## Abbreviations

APACHE: Acute Physiology and Chronic Health Evaluation
NA: Noradrenaline
RASS: Richmond agitation-sedation scale

## References

[1] Cioccari L, Luethi N, Bailey M, Shehabi Y, Howe B, Messmer AS (2020). The effect of dexmedetomidine on vasopressor requirements in patients with septic shock: a subgroup analysis of the Sedation Practice in Intensive Care Evaluation [SPICE III] Trial. Crit Care.

[2] Pichot C, Mathern P, Khettab F, Ghignone M, Geloen A, Quintin L (2010). Increased pressor response to noradrenaline during septic shock following clonidine?. Anaesth Intensive Care.

[3] Leroy S, Aladin L, Laplace C, Jalem S, Rosenthal J, Abrial A (2017). Introduction of a centrally anti-hypertensive, clonidine, reduces noradrenaline requirements in septic shock caused by necrotizing enterocolitis. Am J Emerg Med.

[4] Morelli A, Sanfilippo F, Arnemann P, Hessler M, Kampmeier TG, D'Egidio A (2018). The effect of propofol and dexmedetomidine sedation on norepinephrine requirements in septic shock patients: a crossover trial. Crit Care Med.

[5] Shehabi Y, Howe BD, Bellomo R, Arabi YM, Bailey M, Bass FE (2019). Early sedation with dexmedetomidine in critically Ill patients. N Engl J Med.

[6] Lankadeva YR, Ma S, Iguchi N (2019). Dexmedetomidine reduces norepinephrine requirements and preserves renal oxygenation and function in ovine septic acute kidney injury. Kidney Int.

[7] Lankadeva YR, Booth LC, Kosaka J (2015). Clonidine restores pressor responsiveness to phenylephrine and angiotensin II in ovine sepsis. Crit Care Med.

[8] Morelli A, Sanfilippo F, Arnemann P (2019). The effect of propofol and dexmedetomidine sedation on norepinephrine requirements in septic shock patients: a cross over trial. Crit Care Med.

[9] Pichot C, Mathern P, Khettab F (2010). Increased pressor response to noradrenaline during septic shock following clonidine. Anaesth Intensive Care.

