# Supplementary material for: Septic shock, noradrenaline requirements and alpha-2 agonists: Fishing in the right pond?
Source: Crit Care. 2021 Feb 9;25:56. doi: 10.1186/s13054-020-03377-5 (PMC7874487; doi:10.1186/s13054-020-03377-5)
Supplement: Related texts

# Reply: Authors’ response

**Authors:** Luca Cioccari, Rinaldo Bellomo

**Affiliations:** 3 grid.5734.5 0000 0001 0726 5157 Department of Intensive Care Medicine, Inselspital, Bern University Hospital, University of Bern, Bern, Switzerland; 4 grid.1002.3 0000 0004 1936 7857 Australian and New Zealand Intensive Care Research Centre, School of Public Health and Preventive Medicine, Monash University, Prahran, VIC 3004 Australia; 5 grid.1008.9 0000 0001 2179 088X Centre for Integrated Critical Care, The University of Melbourne, Melbourne, Australia

We thank Dargent and colleagues for the comments about our study on the hemodynamic changes seen in patients with septic shock treated with dexmedetomidine as primary sedating agent versus usual care [1]. We agree that the retrospective nature of the study creates problems because the original study was a sedation trial with mortality as the outcome, while the retrospective investigation of the hemodynamic effects of dexmedetomidine was a post hoc physiological assessment in patients who were mostly deeply sedated at the time of investigation (median RASS of − 3) and receiving propofol in most cases, fentanyl in the majority and midazolam in close to half of cases. In such a setting, the effect of dexmedetomidine is markedly attenuated by the impact of these drugs. Thus, we agree that it is all the more remarkable that, in the dexmedetomidine group, the overall norepinephrine (noradrenaline) requirements were lower and that the dose required to achieve target mean arterial pressure was also decreased. Finally, we agree that comparing patients on dexmedetomidine only versus patients receiving usual care would be ideal. Unfortunately, we were unable to identify such a cohort of patients. Nonetheless, within the limitations of the design and the population studied, we think that our findings are consistent with a substantial body of experimental data supporting the view that, in the septic, vasodilated state, dexmedetomidine (and central alpha-2 agonists) infusion does not exacerbate hypotension or increase vasopressor requirements but, in fact, appears to do the opposite [6–9].
